# Retinal progenitor cells release extracellular vesicles containing developmental transcription factors, microRNA and membrane proteins

**DOI:** 10.1038/s41598-018-20421-1

**Published:** 2018-02-12

**Authors:** Jing Zhou, Alberto Benito-Martin, Jason Mighty, Lynne Chang, Shima Ghoroghi, Hao Wu, Madeline Wong, Sara Guariglia, Petr Baranov, Michael Young, Rajendra Gharbaran, Mark Emerson, Milica Tesic Mark, Henrik Molina, M. Valeria Canto-Soler, Hector Peinado Selgas, Stephen Redenti

**Affiliations:** 1 Department of Biological Sciences, Lehman College, City University of New York, 250 Bedford Park Boulevard West, Bronx, NY 10468 USA; 2 Biology Doctoral Program, The Graduate School and University Center, City University of New York, 365 5th Avenue, New York, NY 10016 USA; 3 Children’s Cancer and Blood Foundation Laboratories, Departments of Pediatrics, and Cell and Developmental Biology, Drukier Institute for Children’s Health, Meyer Cancer Center, Weill Cornell Medical College, New York, New York 10021 USA; 4 Nikon Instruments Inc, 1300 Walt Whitman Road, Melville, NY 11747 USA; 5 Department of Environmental Health Sciences, Mailman School of Public Health, Columbia University, 722 West 168th St, New York, NY 10032 USA; 6 The Schepens Eye Research Institute, Massachusetts Eye and Ear, Harvard Medical School, 20 Staniford Street, Boston, MA 02114 USA; 7 Department of Biology, The City College of New York, City University of New York, New York, NY 10031 USA; 8 Proteomics Resource Center, The Rockefeller University, 1230 York Avenue, New York, NY 10065 USA; 9 The Wilmer Eye Institute, Johns Hopkins University School of Medicine, Baltimore, MD 21287 USA; 10 Microenvironment and Metastasis Laboratory, Department of Molecular Oncology, Spanish National Cancer Research Centre (CNIO), Melchor Fernández Almagro, 3, Madrid, E28029 Spain; 11 Biochemistry Doctoral Program, The Graduate School and University Center, City University of New York, 365 5th Avenue, New York, NY 10016 USA

## Abstract

A range of cell types, including embryonic stem cells, neurons and astrocytes have been shown to release extracellular vesicles (EVs) containing molecular cargo. Across cell types, EVs facilitate transfer of mRNA, microRNA and proteins between cells. Here we describe the release kinetics and content of EVs from mouse retinal progenitor cells (mRPCs). Interestingly, mRPC derived EVs contain mRNA, miRNA and proteins associated with multipotency and retinal development. Transcripts enclosed in mRPC EVs, include the transcription factors Pax6, Hes1, and Sox2, a mitotic chromosome stabilizer Ki67, and the neural intermediate filaments Nestin and GFAP. Proteomic analysis of EV content revealed retinogenic growth factors and morphogen proteins. mRPC EVs were shown to transfer GFP mRNA between cell populations. Finally, analysis of EV mediated functional cargo delivery, using the Cre-loxP recombination system, revealed transfer and uptake of Cre+ EVs, which were then internalized by target mRPCs activating responder loxP GFP expression. In summary, the data supports a paradigm of EV genetic material encapsulation and transfer within RPC populations. RPC EV transfer may influence recipient RPC transcriptional and post-transcriptional regulation, representing a novel mechanism of differentiation and fate determination during retinal development.

## Introduction

A growing number of studies are defining a novel form of cell-to-cell communication involving genetic material exchange via secreted extracellular vesicles (EVs)^1–3^. EVs include exosomes and microvesicles, which are lipid enclosed cell fragments with diameters ranging from approximately 30 nm to 1 μm, released from most cell types studied including cancer cells, embryonic stem cells, hematopoietic stem cells, neurons and astroctytes^4–8^. Exosomes have diameters of 30–150 nm and are formed through the endosomal-sorting complex required for transport (ESCRT) machinery^9,10^. Microvesicles range in diameter from 100–1000 nm and are formed by membrane budding mediated by interactions between cell wall cytoskeletal and phospholipid proteins^11,12^. The release of microvesicles are correlated to cytoplasmic calcium levels and signaling pathways involved in plasma membrane remodeling^13^. Comprehensive EV analysis has been performed on several bodily fluids, including blood, saliva, urine, cerebral spinal fluid^14^ and breast milk^15,16^. Across studies, EVs enclose cytoplasmic and lipid bilayer embedded molecules, leading to encapsulation of unique combinations of microRNA, mRNA and proteins similar to those present in the cells from which they originate^17^. DNA has been reported in EVs from tumor cells, which carry single- and double stranded DNA, retrotransposon elements, and amplified c-Myc oncogene sequences^18^. EVs derived from astrocytes have also been shown to contain mitochondrial DNA^19^.

Recently, oligodendrocyte derived exosomes have been shown to contain molecular cargo that can be functionally recovered in neurons, enhancing neuronal viability^20^. EVs from human embryonic stem cells (hESCs) are capable of reprogramming hematopoietic progenitors through transfer of oct-4, nanog and gata-4^21,22^, suggesting a larger yet to be defined role for EVs in pluripotency, progenitor proliferation and fate determination^22^. EVs derived from hESCs and iPSCs contain a range of microRNAs, suggesting a potential role of EVs in post-transcriptional regulation^17^. Similarly, by transfer of mRNAs and proteins, EVs released from adult progenitor cells in kidney, lung and liver, induce de-differentiation of differentiated resident cells into stem cell-like phenotypes, leading to activation of regenerative programs^1,23^.

Additional studies have described functional effects of adult neuron and neural progenitor EV signaling in differentiation and physiology^8,24,25^. Huttner *et al*.^26^ reported that during neurogenesis in developing mouse brain, neural progenitor derived EVs contain stem cell makers including prominin-1, with predicted involvement in proliferation regulation. Neural progenitor exosomes contain a high level of miRNA-128b and have been shown to direct neuronal differentiation in stem cell populations^25^. EVs released from cortical neurons and microglia contain cell-adhesion molecules and glutamate receptors with predicted function in synapse formation^8,26,27^. Emerging studies of EV content and function in the nervous system suggest potentially important roles in neurogenesis and physiology.

During retinal development, multipotent RPCs are enriched in early transcription factors and miRNAs, which are differentially expressed, influencing fate determination^28–30^. During retinogenesis, multipotent mRPCs pass through a series of competence states, generating subsets of retinal cell types with signature expression profiles. In a conserved temporal sequence, early mouse retinogenesis (embryonic day 8–14) generates ganglion, cone, horizontal, and amacrine cells. In late retinogenesis (embryonic day 16 to postnatal day 12) bipolar, rod, and Muller cells are born. According to this competence model, the progeny of RPC fate is regulated by a combination of extrinsic and intrinsic cellular signaling, yet to be fully characterized^31,32^.

In this work, we demonstrate that mRPCs release EVs with molecular cargo reflective of the expression states of the releasing cells. Our results suggest that mRPC EVs encapsulate miRNA, mRNA and proteins involved in multipotency and differentiation. These data support that mRPC EVs are a novel mechanism of cell-cell communication that could potentially be involved in retinal development, regulating gene expression, post-transcriptional modification and fate specification.

## Results

### Extracellular vesicles are consistently released from mRPCs

**Figure 1:**
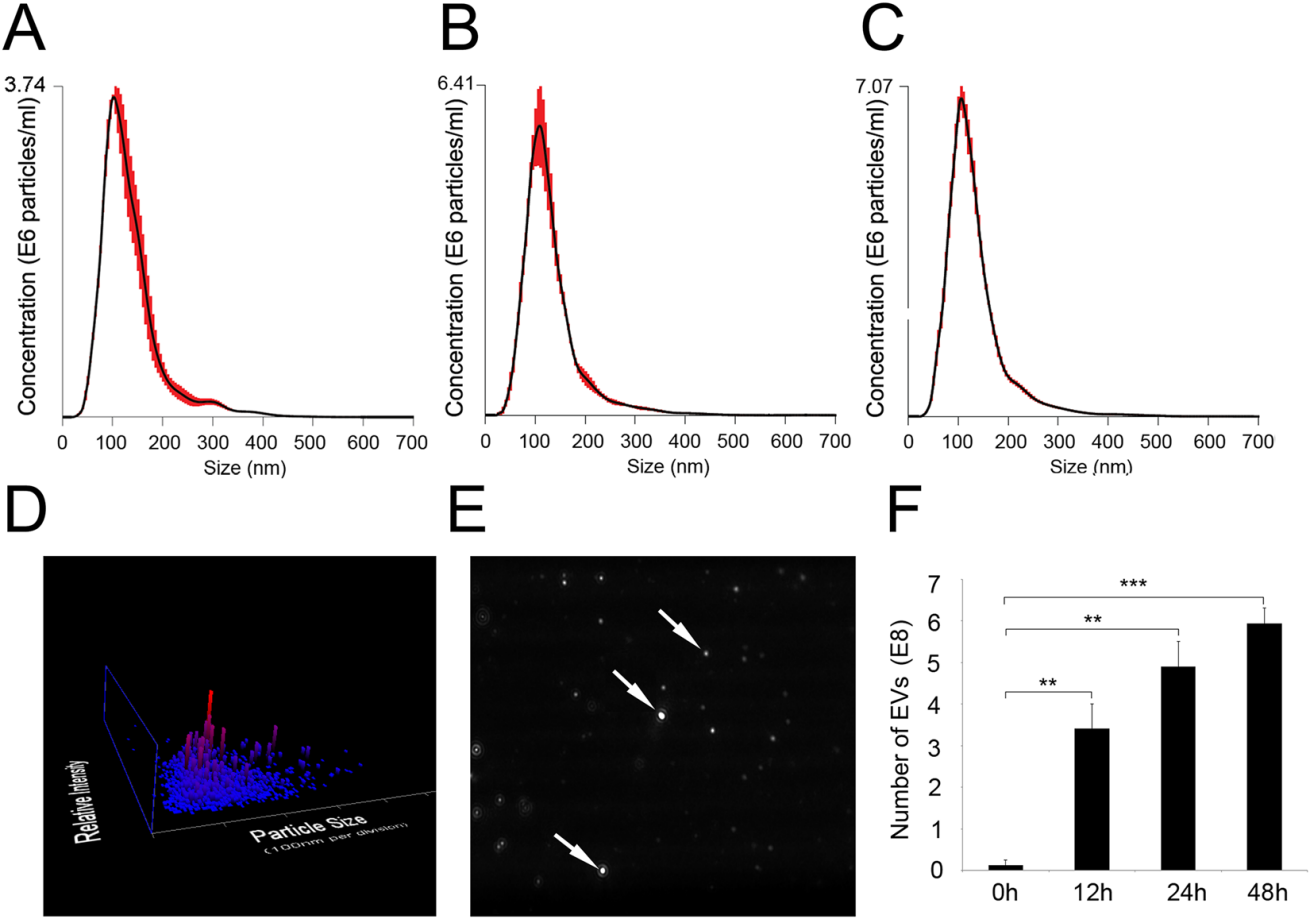
Nanosight analysis of mRPC released extracellular vesicle diameters and concentrations. (**A**–**C**) Nanometer diameter ranges and concentrations of mRPC released EVs were analyzed from conditioned media at 12, 24 and 48 h, respectively. Mean diameter for three samples at each time point analyzed were: 12 h = 133+/−3.2 nm, 24 h = 130+/−0.6 nm and 48 h = 132+/−1.8 nm. (**D**) Sample 3D plot showing EV size/relative intensity at 24 h. (**E**) Arrows showed light-scattering of individual EVs from a single frame of Nanosight tracking analysis at 24 h. (**F**) The average concentration of EVs/ml recorded at 0 h = 0.13+/−0.07 E8, 12 h = 3.41+/−0.34 E8, 24 h = 4.91+/−0.35 E8 and 48 h = 5.93+/−0.22 E8.

In these studies, mRPCs were observed to release EVs consistently over the time course analyzed. The robust viability of mRPCs during the time points of EV analysis was verified using a stable tetrazolium salt WST-based assay (Supplementary Figure S1). The size range and concentrations of EVs released from mRPCs were characterized using nanoparticle tracking analysis (NTA) (Fig. 1). EV mean diameters and concentrations were analyzed from mRPC conditioned media at 12, 24 and 48 h (Fig. 1A–D). Mean diameters for triplicate sample analysis at each time point were 133+/−3.2 nm, 130+/−0.6 nm and 132+/−1.8 nm, respectively. The average diameter of EVs in control media was 127+/−6.6 nm. We next analyzed mRPC released EV concentrations over time. The average concentrations of EVs/ml recorded were 0 h = 0.13+/−0.07 E8 (control), 12 h = 3.41+/−0.34 E8, 24 h = 4.91+/−0.35 E8 and 48 h = 5.93+/−0.22 E8. Data at time 0 h (control) was obtained from fresh media without cells that contained minimal amounts of EVs. After normalization to control, individual mRPCs were found to release approximately 10^4^ EVs within 12 h. A sample of Brownian motion exhibited by mRPC EVs in solution at 24 h is provided in Supplementary Video 1.

### mRPC derived extracellular vesicles exhibit a spheroid morphology and CD63 expression

**Figure 2:**
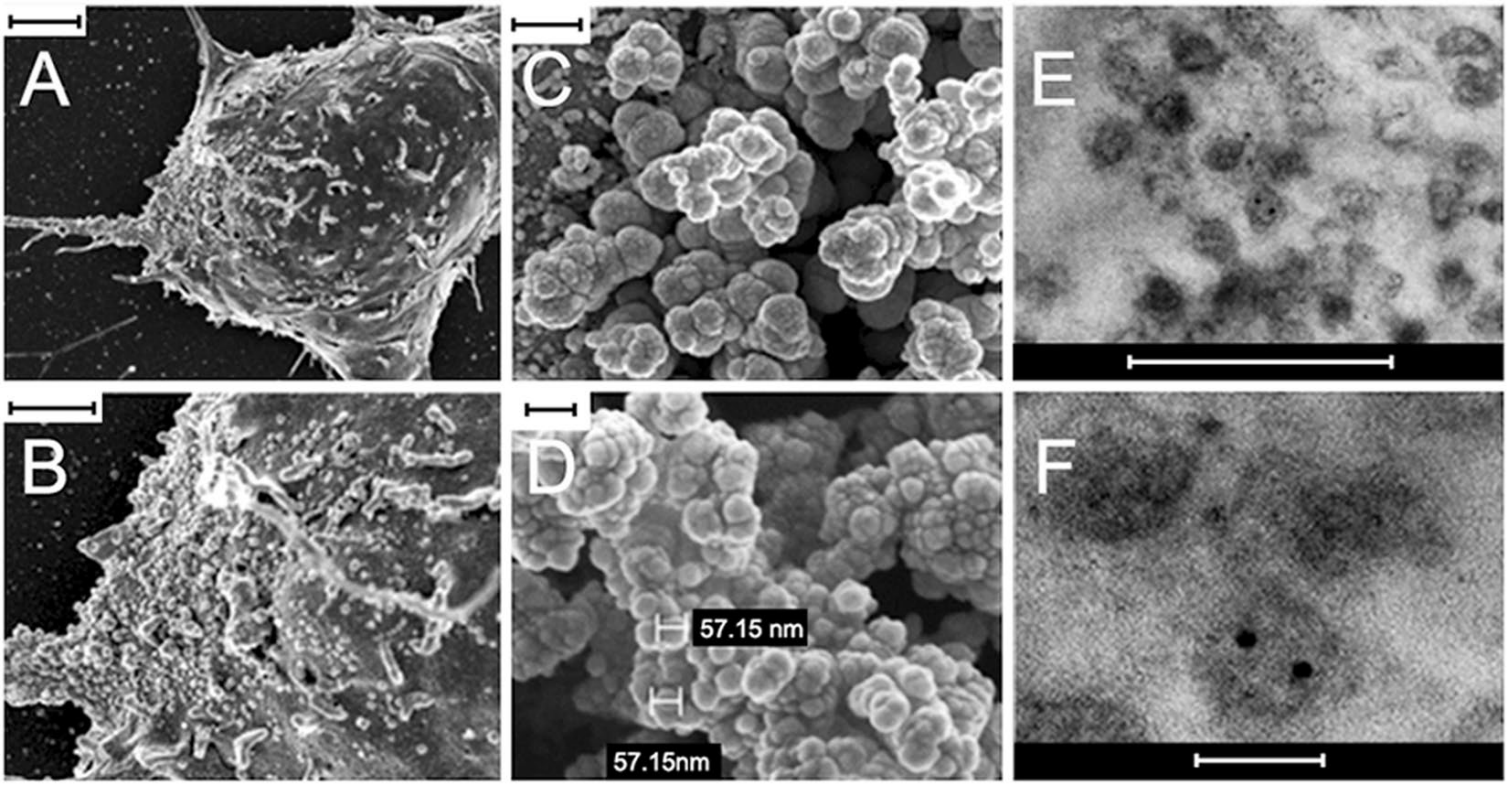
SEM and TEM characterization of mRPC surface and released extracellular vesicle ultrastructure. (**A**) SEM analysis of mRPC ultrastructure with extracellular vesicle structures apparent on the soma and left extended process, scale: 2 μm, (**B**) higher magnification of (**A**), scale: 1 μm. (**C**) released EVs isolated from conditioned media using ultracentrifugation appear as clusters under SEM analysis, scale: 300 nm., (**D**) higher magnification of (**C**) allows for measurement of two vesicles at 57.15 nm, scale: 100 nm. (**E**) Immunogold TEM of isolated EV populations reveal the presence of the EV marker CD63, scale: 500 nm, (**F**) higher magnification of (**E**), scale: 100 nm.

**Figure 3:**
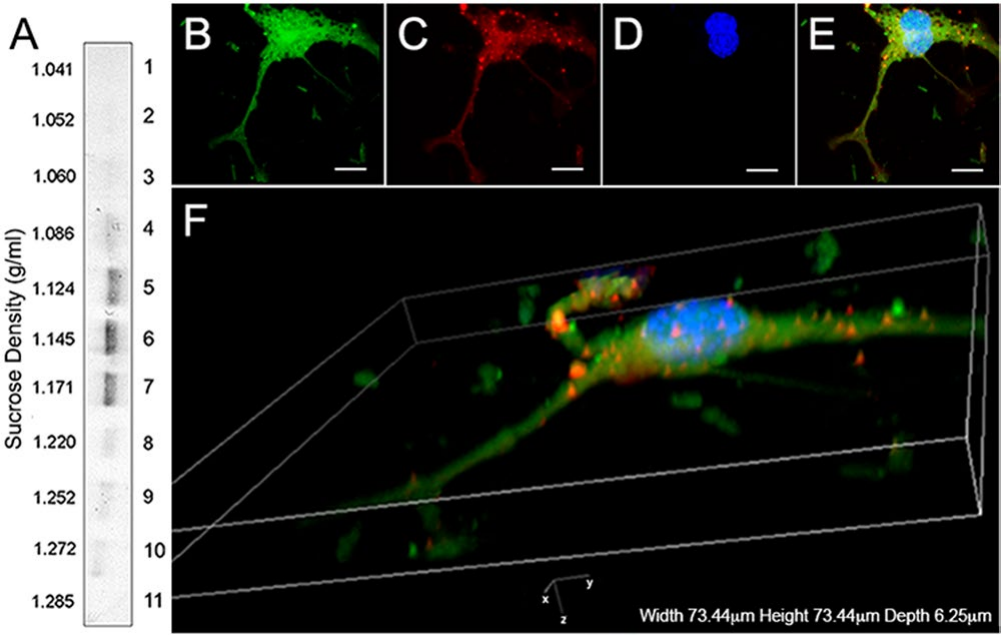
Sucrose-gradient and immunohistochemical characterization of extracellular vesicles. (**A**) CD63 positive bands were detected in fractions at gradient-densities of 1.12–1.17 g/cm^3^. (**B**–**F**) Immunohistochemical analysis EVs: (**B**) GFP+ mRPCs (green), (**C**) anti-CD63 labeling in cytoplasmic and nuclear regions (red), (**D**) DAPI (nuclei, blue) and (**E**) overlay. Scale: 10 µm. (**F**) 3D confocal reconstruction of labeled mRPCs revealed the presence of CD63 positive EVs in the cytoplasm, emerging from lipid bilayer regions of cell soma and on proximal and distal processes.

The size and morphology of isolated mRPC and EV samples were analyzed using scanning electron microscopy (SEM) and transmission electron microscopy (TEM)^33–35^. SEM analysis revealed the presence of EV structures on the soma and proximal processes of mRPCs (Fig. 2A,B). Isolated EVs exhibited self-clustering during SEM analysis, with spheroid morphologies and selected sample diameters of 57.15 nm (Fig. 2C,D). The clustering behavior of EVs may be due to enriched adhesion molecules on EVs. A common feature of many types of EVs is their expression of adhesion molecules, composed of proteins such as IgG superfamily and integrin family, among others. Adhesive properties of EVs are not well known yet^35,36^. Selected images of immunogold TEM analysis showed EV diameters ranging from 70 to 200 nm and positive labeling for the EV marker CD63 (Fig. 2E,F). Next, expression of CD63 was used to characterize predicted EVs present within and emerging from the mRPCs. The tetraspanin CD63 is involved in intraluminal vesicle formation, enriched in EV membranes and a classic marker of EVs^33,37^. Isolated EVs were fractionated on a sucrose-density gradient by ultracentrifugation (Fig. 3). CD63 positive bands were detected using sucrose gradient centrifugation followed by Western blotting in fractions at gradient-densities of 1.12–1.17 g/cm^3^, which aligned well with other published studies characterizing EVs (Fig. 3A)^38,39^. An image of the entire sucrose gradient gel used to generate Fig. 3A appears in Supplementary Figure S4. To visualize CD63 positive EVs present in and emerging from mRPCs, anti-CD63 immunohistochemical analysis was performed. Confocal imaging and 3D reconstruction revealed CD63 labeling in the cytoplasmic spaces of mRPCs as well as on EVs emerging from lipid bilayer regions of both cell soma and processes (Fig. 3C–F).

**Figure 4:**
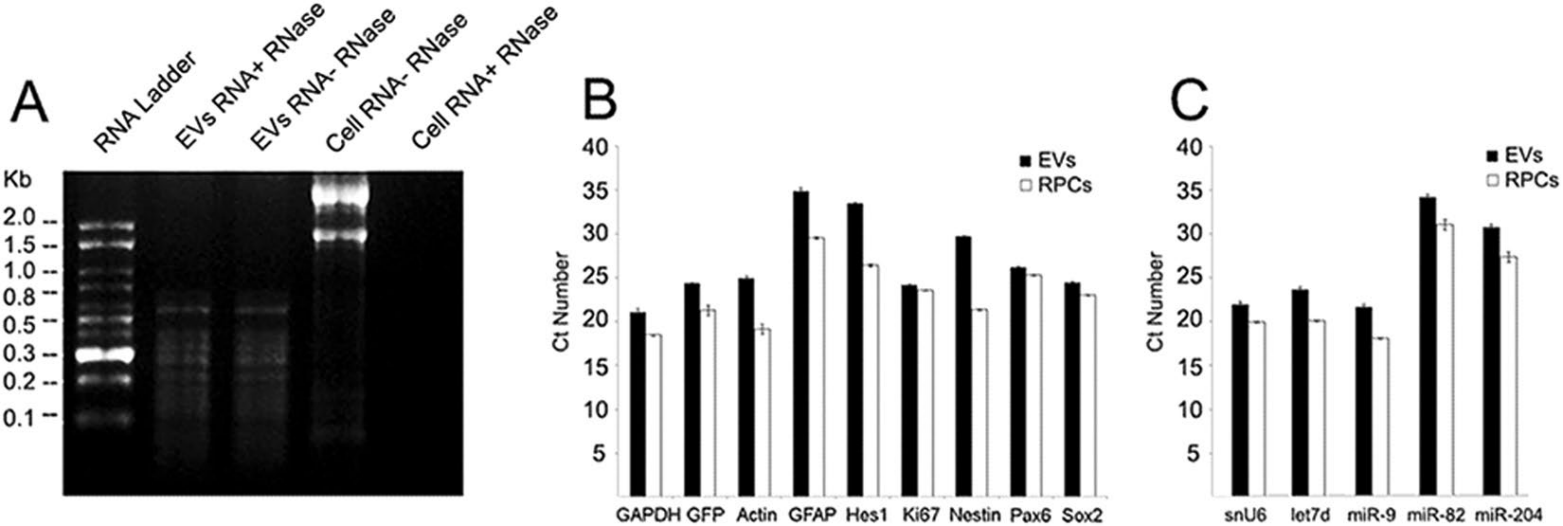
Genetic cargo of mRPC derived extracellular vesicles. (**A**) A 1.5% denaturing agarose gel loaded with total RNA from mRPCs and EVs. Total RNA from EVs consisted primarily of species below 800 nucleotides (nt) lacking 28S and 18S rRNA. EVs were treated with RNase and no difference was detected when compared with non-treated EVs, indicating the RNA of EVs was enclosed within the vesicle membrane. (**B**) Transcription factors, a cell-cycle regulator and intermediate filaments were identified in both mRPCs and EVs included Pax6, Hes1, Sox2, Ki67, GFAP and Nestin. The transcription factors identified are collectively involved in facilitating mRPC multipotency, cell-cycle and fate specification during retinogenesis. GFP, GAPDH and β-actin mRNAs were also detected in mRPCs and EVs. Next, the presence of miRNAs with established expression and function during retinogensis were chosen for analysis. (**C**) Selected miRNA species analyzed included Let7d, miR-9, miR-182 and miR-204. U6 snRNA was used as control. Data presented were combined from four independent replicates.

Extracellular vesicles from a range of cell types have been shown to transfer combinations of mRNA, microRNA, and proteins^40–42^. In this work, following the isolation of EVs from mRPC conditioned media, we analyzed mRNA, miRNA and protein content. Total RNA identified from EVs consisted primarily of species below 800 nucleotides (nt) lacking 28S and 18S rRNA (Fig. 4A). EVs were treated with RNase and no difference was detected between non-treated EVs and treated EVs, indicating the RNA was enclosed within the vesicle membrane (Fig. 4A).

### Extracellular vesicles encapsulate mRNA and miRNA expressed in mRPCs

QPCR results suggested that mRNA species contained in EVs from mRPCs reflect genetic expression patterns of the cells. In particular, identified transcription factor and neuron progenitor cell mRNAs in both mRPCs and EVs included Pax6, Hes1, Ki-67, Sox2 and Nestin (Fig. 4B). qPCR was also performed on EV-depleted control medium to confirm the absence of mRPC transcription factor mRNA or housekeeping genes. The presence of transcription factors in nervous system extracellular vesicles has been described in normal physiological and pathological conditions^43–45^. Pax6, Hes1, Sox2 and Ki67 are involved in multipotency, fate specification and cell-cycle in mRPCs throughout retinal development^28,46^. To compare mRNA levels between EVs and mRPCs, the same amount of starting RNA was used for each qPCR experiment. The controls β-actin and GAPDH are present at higher levels in mRPCs compared to EVs using the same amount of starting mRNA for reverse transcription. Differences in transcript levels between mRPCs and EVs are represented using Ct values as in previous EV studies^3,21,47^. The transcription factors, Ki67 and intermediate filaments analyzed, as well as GFP mRNA, appeared in slightly higher levels in mRPCs when compared to EVs (Fig. 4B). These data revealed that secreted EVs contained a collection of mRNA transcripts important for mRPC multipotency.

The results from agarose gel electrophoresis revealed that the RNA species encapsulated in mRPC EVs were below 800nt, with a percentage appearing below 100nt. To begin to characterize the small RNAs, qPCR was used to investigate the presence of miRNAs in both mRPCs and EVs. Four miRNAs, with established expression and studied function in retinal development, were chosen for analysis, including Let7d, miR-9, miR-182 and miR-204^48–50^. U6 snRNA was used as a stable endogenous control^51^. Each small miRNA species tested appeared in higher levels in mRPCs, with lower detectable levels present in EVs (Fig. 4C).

### Extracellular vesicles are enriched for protein species expressed in mRPCs

**Figure 5:**
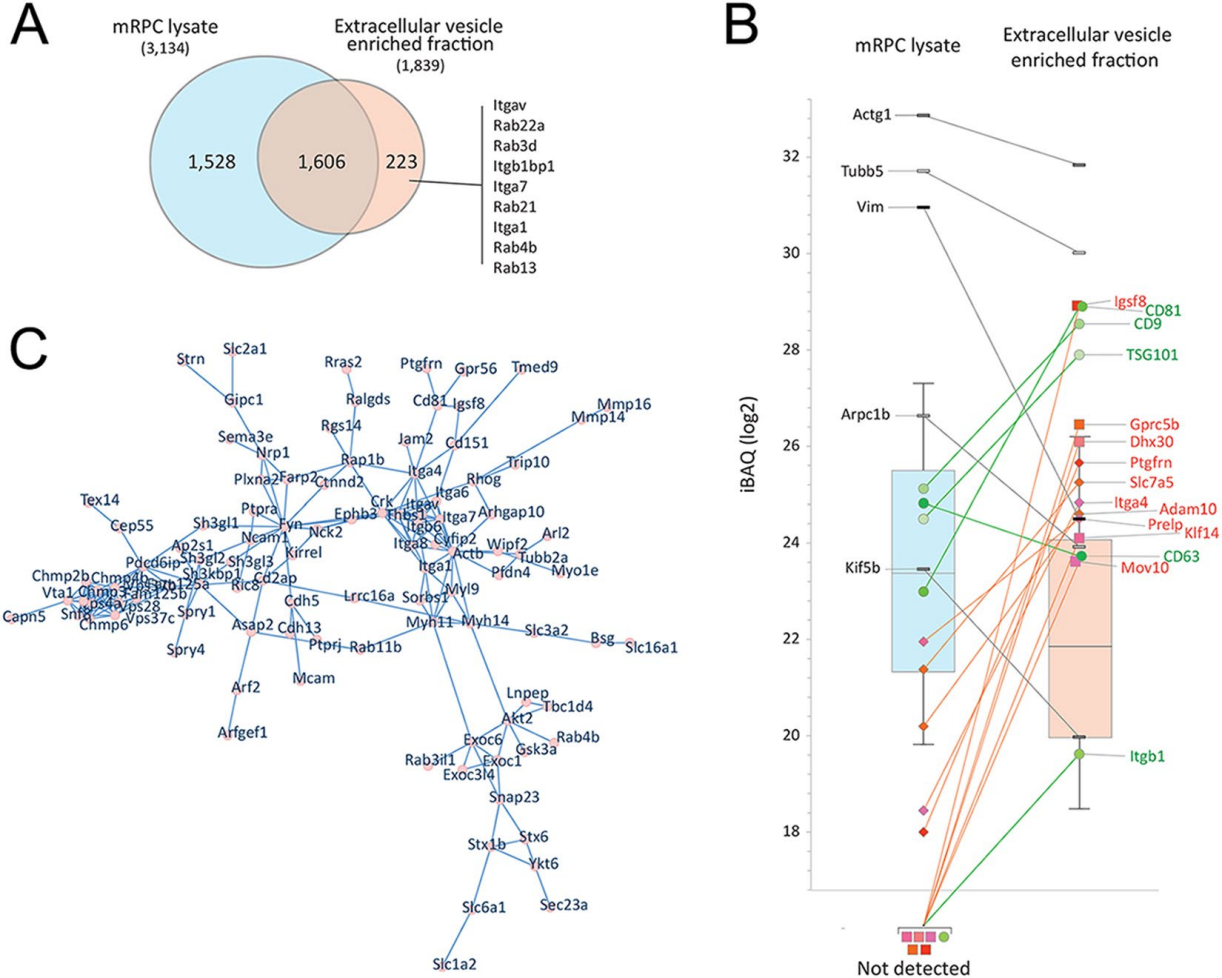
Qualitative proteomic evaluation of extracellular vesicle enriched fractions from mRPCs. (**A**) Venn diagram of unique and shared proteins measured in mRPCs (3,134 - blue) and the enriched EV fractions (1,829 - red). Proteins only measured in the EV enriched samples included Rab family proteins. (**B**) The distribution of iBAQ (intensity Based Absolut Quantitation) values of proteins matched in the two experiments. The measured median iBAQ signal is ~4-fold lower in the EV enriched fraction. Onto the Box Plot are mapped selected proteins belonging to 2 groups: Enriched/unique to EV (green and red) and not-enriched proteins (black/grey). Green indicates proteins often used as EV markers. Red is used for proteins with relevance to mRPC state and development. Squares are used for proteins not identified in the mRPC lysate, while diamonds indicate proteins matched in the mRPC lysate but that are highly enriched in the EV fraction. Black/grey bricks were used to map cellular proteins often observed because of their high abundance. (**C**) STRING-db (high confidence, 0.700) analysis presented using CytoScape^123^. A. selected network of proteins enriched 8-fold or more are shown. This network contains integrins, Rab GTP family, SLC transporters family and other vesicle related proteins.

Liquid chromatography-tandem mass spectrometry (LC-MS/MS) was used to profile the proteome of mRPCs derived EVs^52^. Overall, 1,829 proteins were matched at 1% False Discovery Rates (protein and peptide) in a ‘bottom-up’ proteomics profiling of EV enriched fraction. 3,134 proteins were matched analyzing mRPC lysate. 223 of the proteins matched in the EV enriched sample were not matched in the cell lysate (Fig. 5A). The measured median iBAQ signal was ~4-fold lower in the EV enriched fraction (Fig. 5B). Using the protein iBAQ distribution as a reference, five proteins, established as EV markers, were highly enriched in EVs, including CD81, CD9, TSG101, CD63 and Itgb1 (green circles) (Fig. 5B), consistent with previous reports^53^. Analysis of mRPC derived EVs by LS-MS/MS revealed many common and previously described EV proteins such as cytoskeleton component (actins, myosins, actin interacting regulatory proteins cofilins and tubulins), heat shock proteins (HSP 90-beta, HSP 90-alpha and HSP a4), G-proteins (Gprc 5b), a great number of Rab GTPase associated with membrane transport and fusion and the tetraspanin families’ (Tspan4, Tspan7, Tspan14, Tspan9 and Tspan6).

Rab GTPase family proteins were also present in mRPC derived EVs (Supplementary Table 1). Rab GTPases participate in multivesicular body (MVB) fusion and microvesicle genesis^54^. Two Rab GTPases identified in EVs were Rab11 and Rab35. Rab11 plays a vital role in docking and fusion of MVBs^55^. Rab35 functions in MVB docking and subsequent release of EVs^33^. Our data showed that though Rab35 was measured in both samples, the Rab35 signal was highly enriched in the EV sample (Supplementary Table 1). Additional biogenesis-related proteins identified included vesicle assembly proteins and vesicle transport (Annexins), vacuolar proteins sorting-associated proteins (Snf8, Vps37c, Vps28, Vps4a, Vps36, Vps25 and Vps4b, and signal transduction proteins such as 14-3-3 and Sdcbp proteins. ESCRT proteins identified included ESCRT-0, ESCRT-I, ESCRT-II and ESCRT-III, each with roles in biogenesis of MVBs and release of exosomes. In total, eighteen proteins, mostly vacuolar sorting-associated proteins, identified in this work, belonged to the ESCRT sorting machinery (Supplementary Table 2). Submitting proteins enriched 8-fold or more according to a String analysis^56^ (background proteome: mouse) revealed that indeed, ‘extracellular vesicles and exosomes’, ‘membrane-bound vesicles’ and ‘vesicles’ (GO: 1903561, 0070062, 00319888 and 0031982) were highly enriched (FDR less than 1e-70) among the enriched proteins. Figure 5C shows a network of enriched EV proteins, including integrin, a protein family, which was recently related to exosome structure^57^ and vacuolar protein sorting. In Fig. 5C, the proteins in the network are enriched 8-fold or more in EVs than in cells. This network contained integrin proteins including itga4, itga6, itgb6, itgb1 which were enriched in exosomes, in addition to itga1, Itgav and itga7 which are found exclusively in exosomes. Integrins are a complex family of cell adhesion receptors mediating signaling of cell-cell and cell-extracellular matrix (ECM) protein interactions. Enriched integrins in EVs may mediate self-clustering properties of EVs, as visible in SEM results (Fig. 2A). Integrin α and β subunits form approximately 24 known pairs of heterodimer receptor complexes, which determine ECM cell binding sites^58^. Recently, Itgav was found to mediate multipotent adipose-derived stem cell interactions with surrounding environment and in cell cycle progression. In addition, Itgav is implicated in neural invasion, by malignant tumor cells^59^ and angiogenesis^60,61^. Also, a number of solute carrier (SLC) transporter members were found in EVs, including Slc1a2, Slc2a1, Slc6a1, Slc7a5, Slc12a7, Slc12a2 and Slc16a1 (Fig. 5C and Supplementary Table 4).

In addition to EV associated proteins, several proteins potentially associated with mRPC state and neural development, were identified from proteomic analysis. According to the iBAQ signal, Igsf8, Gprc5b, Dhx30, Klf14 and Mov10 were identified in EVs but not in the mRPC lysate; proteins including Ptgfrn, Slc7a5, Adam10 and Prelp were matched in mRPC lysate but enriched in the EVs (Fig. 5B). Adam10 is involved in maintaining progenitor cell pools and directing neural differentiation by regulating Notch signaling^62,63^. Additionally, Krueppel-like factor 14 (Klf14) is associated with positive regulation of cell cycle and regulation of transcription^64,65^. Additional EV proteins, with predicted involvement in mRPC fate and neural differentiation are provided in Supplementary Table 3. GFP under the control of ubiquitous CAG was also found in both EVs and mRPCs. According to proteomic analysis, the signal of GFP is approximately 20 times lower in EVs than mRPCs (data not shown). A full proteomic data set is provided in Supplemental Table S7.

### Extracellular vesicles released from mRPCs are taken up by target cells

**Figure 6:**
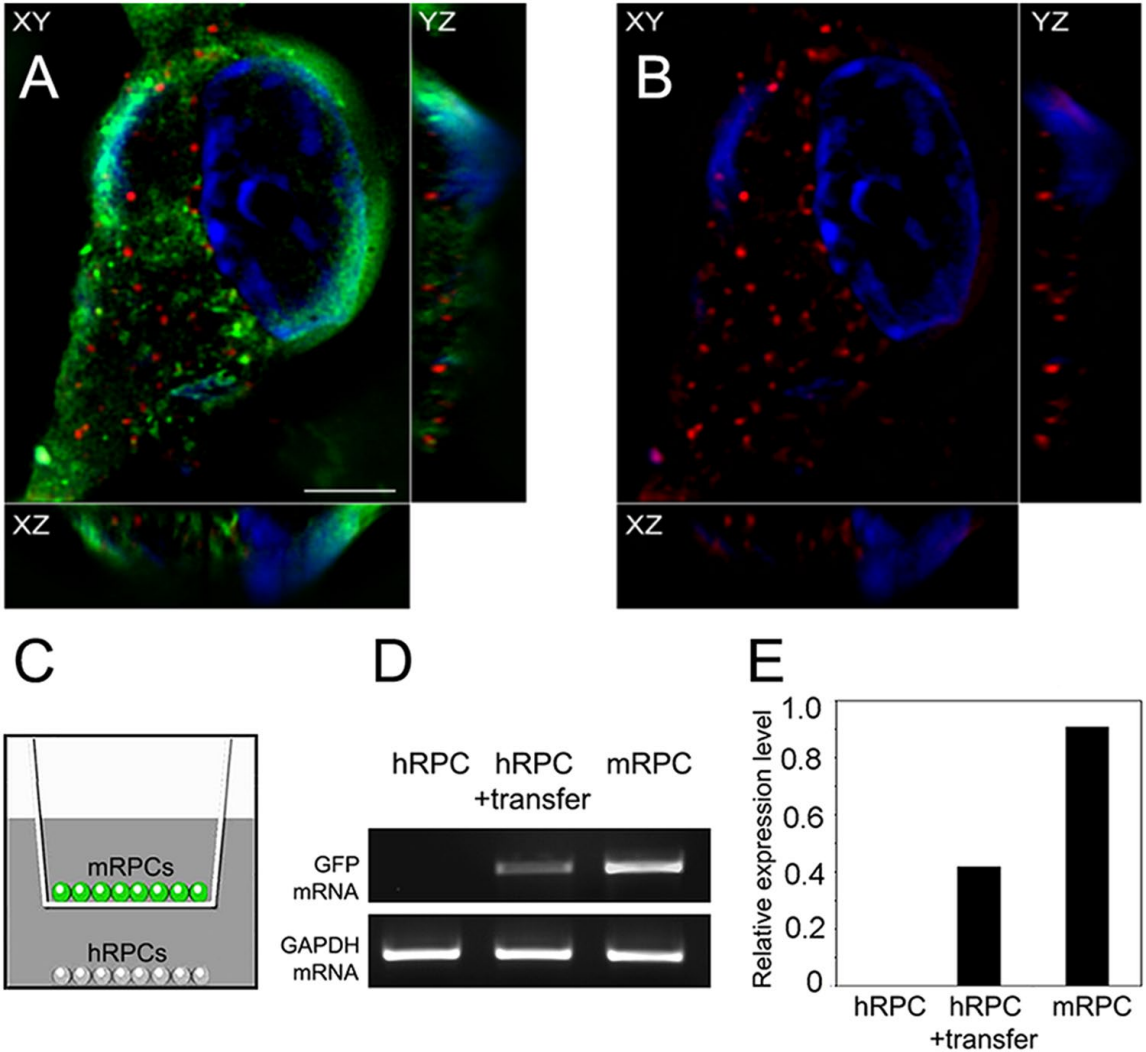
Extracellular vesicle internalization and transfer of GFP mRNA (**A**) Super resolution 3D reconstruction of GFP+ mRPC following 24 h incubation with PKH26 labeled extracellular vesicles. Red vesicles are visibly localized near the cell surface and within cytoplasm. In the XZ axis, GFP (green), EVs (red) and nuclei (blue, DAPI). (**B**) same as (**A**) with GFP (FITC) channel removed to increase visibility of PKH26 (TRITC) labeled EVs. Each panel contains three cross-sectional views (xy, xz, and yz). Scale: 5 µm. (**C**) RT-PCR analysis of GFP mRNA transfer between GFP+ mRPCs and non-GFP hRPCs. Non-GFP hRPCs served as negative control; GFP+ mRPCs served as postive control. GAPDH served as the internal control gene. EVs were treated using an RNase-Free DNase Set to remove DNA comtamination before cDNA synthesis. (**D**) Intensities of RT-PCR images were measured with ImageJ software and normalized to GAPDH. Relative levels of hRPC GFP after transfer of EVs is significantly higher than negative control.

EVs isolated from conditioned media were taken up by recipient mRPCs. For initial extracellular vesicle uptake analysis, isolated EVs, stained with PKH26, were incubated with live mRPCs for 24 h, followed by fixation and microscopic imaging. A low power image of mRPCs with PKH26 labeled EVs is provided as Supplementary Figure S2. For high power analysis of EV cell surface binding and internalization in recipient mRPCs, 3D super-resolution microscopy was utilized. 3D reconstruction with three channels (XY, XZ, YZ) overlayed, revealed that EVs were localized to the lipid bilayer, internalized to the cytoplasmic space and potentially to the nucleus of mRPCs (Fig. 6A,B). In additon, mRPC internalized PKH26 labelled EVs revealed co-localization with the EV marker CD63 (Supplementary Figure S3). Internalized EVs have been shown to be transported along microtubules, localized within the perinuclear region and nuclear envelope^66,67^.

### Extracellular vesicles transfer encapsulated mRNA to target cells

Next, the transfer of GFP mRNA from GFP+ mRPC EVs to non-GFP hRPCS was analyzed using co-culture and RT-PCR. GFP+ mRPCs and hRPCs were co-cultured for 96 hrs, while divided by a transwell mebrane containing 0.4 µm pores. Total RNA from hRPCs without mRPC transfer was used as a negative control. RT-PCR was performed to analyze if GFP mRNA was transferred to recipient cells by EVs. Results showed no GFP signal in the negative control group (Fig. 6C). Following RT-PCR result showed that GFP mRNA was transferred to hRPCs within the 96 h period (Fig. 6C,D). The data reveals EV mediated transfer of GFP mRNA and supports the possibility that transcription factors and miRNA identified in EVs may also be transferred between mRPCs.

### Extracellular vesicles transfer functional Cre to reporter loxP mRPCs

**Figure 7:**
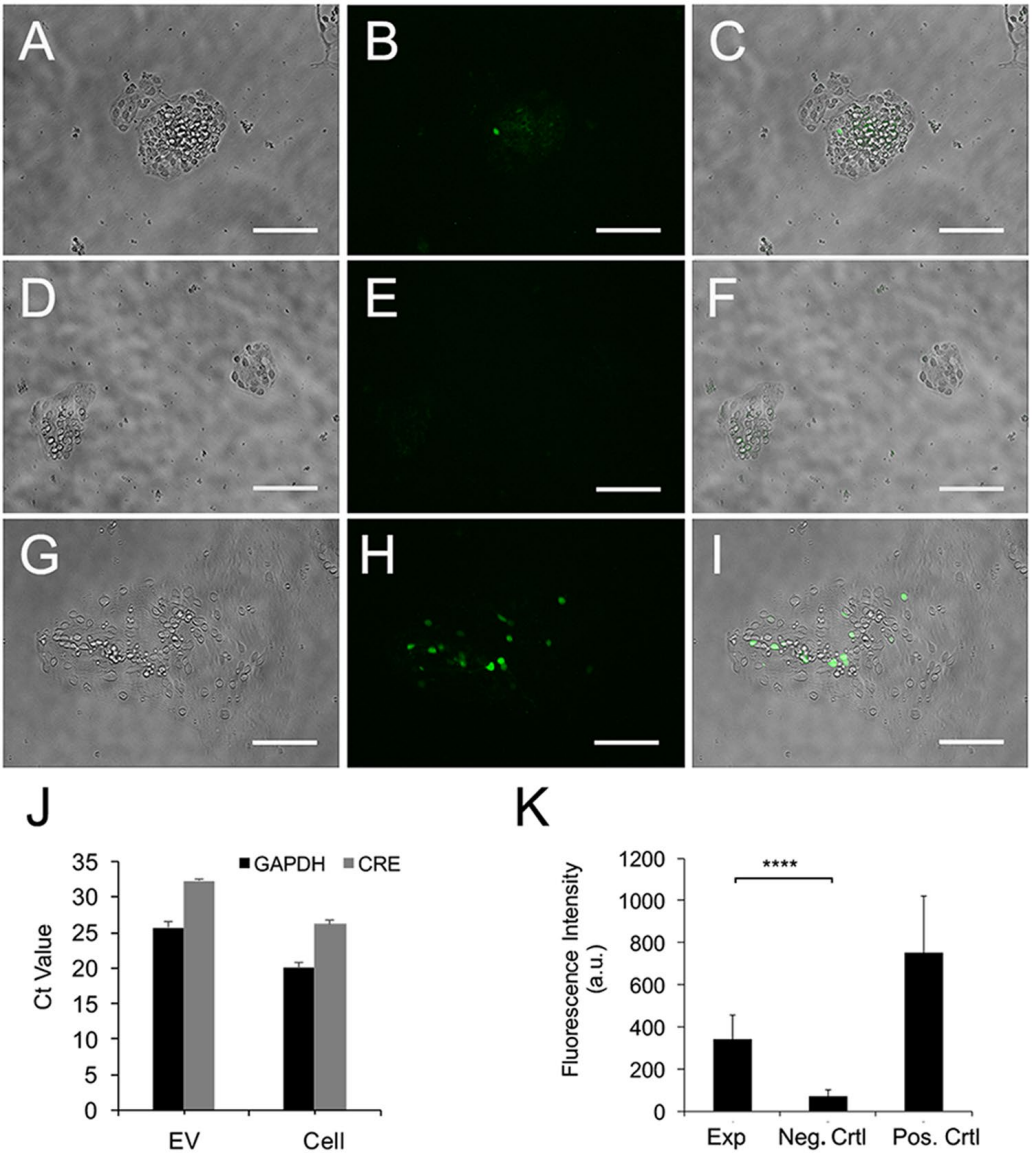
Extracellular vesicle functional transfer of Cre in dissociated mRPCs. P0 retina were electroporated with Cre+ plasmid, dissociated into mRPCs and plated in 0.4 µm pore transwell inserts. Dissociated mRPCs electroporated with reporter constructs were placed in lower wells beneath Cre+ mRPCs. (**A**–**C**) EV mediated functional transfer of Cre leading to activation of GFP expression in reporter RPCs as can be observed in experimental wells following 14 days of culture. (**D**–**F**) Negative controls, containing reporter constructs electroporated mRPCs, did not show GFP expression. (**G**–**I**) Positive controls, electroporated with both Cre+ and reporter constructs, showed robust GFP expression. Scale: 100 µm. (**J**) Real time PCR verified Cre+ signal in EV derived from Cre+ electroporated cells. (**K**) Analysis of GFP intensity in mRPCs revealed a significant difference between experimental and neg. control (experimental: mean 341.41, sd115.92, neg.control mean 70.39 sd 31.65) P value < 0.0001 experimental vs. negative control and between experimental and pos.control (751.42 sd 271.1293979) P value = 0.05 experimental vs positive control.

pCAG-Cre plasmid containing CMV enhancer and chicken beta-actin promoter was used as Cre expression vector; pCALNL-GFP containing chicken beta-actin promoter and EGFP sequence was used as reporter vector^68^. P0 mouse retina electroporated with both pCAG-Cre and pCALNL-GFP plasmids, were dissociated into mRPCs and used as positive control. In the experimental group, retinas electroporated with only pCAG-Cre were dissociated and seeded into transwell inserts, while pCALNL-GFP electroporated retinae were dissociated and seeded in the lower wells. Non-electroporated mRPCs in transwell inserts with reporter mRPCs seeded in the lower wells were used as negative control. GFP-positive cells were detected in experimental group starting from day 18 after electroporation (Fig. 7A–C); in contrast, negative control showed limited background without cell specificity (Fig. 7D–F). Analysis of electroporation efficiency in positive controls, indicated that approximately 9% of dissociated mRPCs expressed detectable levels of GFP (Fig. 7G–I). Cre genes in EV and mRPCs were verified by real time PCR (Fig. 7J). Ct values were 32 versus 26 in EVs and mRPCs, respectively; no free floating Cre was detected in supernatant after isolation of EVs (data not shown). Reporter mRPC GFP intensities were measured for experimental, negative control and positive control using ImageJ. The results demonstrated that, compared to negative controls, experimental mRPCs showed significant increases in GFP fluorescence consistent with EV mediated transfer and reporter activation (Fig. 7K).

## Discussion

Multipotent mRPCs are guided by genetic and epigenetic cues during retinal differentiation and fate specification. In addition to cell-autonomous differential gene expression and miRNA mediated post- transcriptional modifications, a number of studies revealed that media conditioned from retinal cells provides soluble cues capable of influencing mRPC fate^69–74^. It is possible that factors secreted from the developing retina, that influence retinogenesis, may be contained in EVs. In this work, mRPC EVs were shown to contain mRNA species associated with transport and transcription including poly(A)-binding protein cytoplasmic 4 (Pabpc4) with demonstrated function in supporting mRNA expression via increased stability and protection from decay^75^. Another identified protein in EVs with potential roles in mRNA transport and transcription is the mediator of RNA polymerase II transcription subunit 23 (Med23), required for transcriptional activation and assembly of the pre-initiation complex^76^.

This work also demonstrates that mRPC EVs contain mRNA of transcripts involved in retinal development. mRPC EVs were observed to contain transcription factors including Pax6, Hes1 and Sox2. Additionally, the miRNAs identified in EVs included Let7d, miR-9, miR-182 and miR-204. Interestingly, the ratio of RNA species in mRPCs and EVs differed. For example, Ki67 showed higher expression compared to other mRNAs in mRPCs, while in EVs, Nestin mRNA was present in higher levels than in mRPCs. The differential distribution of RNA species between mRPCs and EVs may indicate selective packaging of transferable RNA as a mechanism involved in modulating retinogenesis^77,78^. While, a number of molecular mechanisms are suggested to play roles in the differential distribution of RNA species in EVs, including, 3′ adenylation, cell RNA levels and RNA affinities to the MVBs outer surface, the exact process remains to be fully defined^79–81^.

A number of studies demonstrate the presence of functional miRNAs in extracellular spaces and in cerebral spinal fluid^82–85^. Additionally, extracellular miRNAs exhibit resistance to high extracellular ribonuclease (RNase) activity^82^ increasing the probability of packaging in membrane bound EVs. EV mediated miRNA transfer is emerging as an important regulator of cell-to-cell regulation of gene expression in a number of tissue types^2,21^. Accordingly, our data reveals that subsets of miRNAs are present in mRPC EVs, including miRNA9, miRNA182, miRNA204 and Let7d. Analysis of mouse retinal miRNA transcriptome^50^ reveals that miR-9 is highly expressed in neonatal retina, with peak expression near P10; miR-182 interacts with a photoreceptor-specific cluster of genes and increases expression after P1. MiR-204 is expressed in the developing retina during rod photoreceptor differentiation and later in the ciliary margin^48,49^. Let7d plays important roles in neural fate specification, with predicted function in RPC differentiation^86,87^. Changes in expression levels of mRPC EV encapsulated miRNAs may provide a level of cell-to-cell genetic regulation active during retinal development^50^.

EVs derived from mRPCs may also provide protein cargo to facilitate morphogenic processes. Morphogenesis proteins identified in mRPC EVs include, Latrophilin-3 (Lphn3)^88^, which facilitates cell adhesion and synapse formation, Integrin alpha-6 (Itga6)^89^, active in retinal lamination and neurite outgrowth, as well as neural cell adhesion molecule 1 (Ncam1) associated with retinogenesis^90^ (Supplementary Table 3). Potential mediators of mRPC physiology contained in EVs include the Ca^2+^-activated chloride channel, tweety homolog 2 (Ttyh2)^91^, the voltage-dependent calcium channel gamma-7 subunit (Cacng7)^92^, which regulates the trafficking and gating properties of AMPA-selective glutamate receptors and the Monocarboxylate transporter 1 (Slc16a1) known to regulate lactate exchange between neurons and glia, localized in late development in Müller processes and plexiform layers^93^. Additional channel proteins and transport proteins are listed in Supplementary Tables 4 and 5, respectively.

Solute carrier (SLC) transporters are a family of membrane proteins involved in cellular uptake of small molecules. mRPC EVs are enriched in SLC transporter proteins including Slc1a2, Slc2a1, Slc6a1, Slc7a5, Slc12a7, Slc12a2 and Slc16a1 (Fig. 5C and Supplementary Table 4). Slc1a2 is a glutamate transporter, mainly expressed in neurons, involved in removing synaptic glutamate and terminating glutamatergic transmission^94^. Slc2a1 is also involved in uptake of glucose^95,96^. An additional mRPC EV amino acid transporter is ASCT1 (Slc1a4) which facilitates electroneutral exchange of amino acids^97^. Also, lactate transporter Slc16a1 is involved in regulation of lactate transport, which increases during glycolysis^98,99^. In addition, Slc3a2 regulates the function of integrins 5–8 and modulates amino acid transport^100–102^.

In conclusion, data presented in this work provides the first analysis of release rate, morphology, content and transfer of EVs derived from mRPCs. As predicted, the mRNA, miRNA and protein contents of EVs reflect the general expression states of mRPCs, while the ratio difference in levels of mRNA species in EVs compared to mRPCs suggests a possible mechanism of selectivity involved in determining cargo. The transfer of GFP mRNA and Cre+ in mRPC derived EVs supports the possibility that a range of functional mRNA, miRNA and protein species may be transferred in EVs *in vitro and in vivo*. Taken together, the data suggest that mRPC EV cell-to-cell transfer of molecular cargo may present a novel form of genetic regulation and soluble signaling involved in RPC differentiation and retinal development.

## Materials and Methods

### mRPC isolation and culture

All animal procedures were performed in compliance with the Association for Research in Vision and Ophthalmology (ARVO) statement for the use of animals in ophthalmic and vision research, and were approved by the City University of New York, Lehman College Animal Care and Use Committee (IACUC). Actin promoter-GFP multipotent mRPCs (mRPCs) were isolated from post-natal day one mice and culture expanded as previously described^103,104^. Briefly, mRPCs were grown at 37 °C and 5% CO_2_ in neurobasal complete media containing 2 mM glutamate, 1× EV-depleted B27 supplement, 1× N2 supplement, 20 ng/ml recombinant epidermal growth factor (rEGF), 50 ug/ml nystatin, penicillin-streptomycin (10 IU ml-1 and 20 ug ml-1), respectively. For EV isolation, multipotent mRPCs, at passage 15 were grown in T-75 flasks while proliferating and growing as both single cells and neurospheres^104^. EVs were depleted from culture media by first centrifuging at 100,000 × g for 70 min and then filtering using a 0.22 µm pore sized filter.

### Cell viability assay

mRPC suspension was prepared from one T-75 flask at 80% confluency. Cell suspension was then centrifuged at 300 g for 5 min. Cell pellet was re-suspended in 5 ml complete neurobasal media as above. The number of viable cells was counted and plated at the concentration of 1.0*10^5^cells/cm^3^ with 2 ml of media in 24 well plates. Cells were incubated for 0, 24 and 48 h (n = 4 each). After incubation at 0, 24, and 48 h, a viability assay was performed using WST-1 reagent (WST-1, CELLPRO ROCHE). 200ul of WST-1 reagent was added per well and incubated for 2 h before its quantification using a microplate reader (Benchmark Microplate Reader; BIO-RAD) at 450cy5 nm. WST-1 is a colorimetric assay that uses a soluble tetrazolium salt, which is then cleaved to a colored product known as formazan by the reductase system of metabolically live cells.

### Isolation of mRPC EVs

EVs were isolated from media conditioned by 1 × 10^7^ mRPCs cultured in T75 flasks for 48 h. Media was centrifuged at 300 × g for 10 min at 4 °C to pellet cell debris. Briefly, supernatant was transferred to an ultracentrifuge tube (Beckman Coulter), spun at 10,000 × g for 20 min using 60Ti rotor (Beckman Coulter ultracentrifuge) at 4 °C; supernatant was filtered through 0.22 µm filter and centrifuged at 100, 000 × g for 70 min to pellet the EVs^21^. All centrifugation was performed at 4 °C to minimize degradation of EVs. EVs were suspended in phosphate-buffered saline (PBS) and stored at −80 °C for analysis. While ultracentrifugation was used in this work, the authors acknowledge that different EV isolation techniques can affect protein and RNA profiles of isolated EVs^105,106^.

### Scanning electron microscopy (SEM)

EVs were fixed with 2% glutaraldehyde (EM grade) in 0.1 M phosphate buffer, pH 7.2, at room temperature for 1 h. Samples were then rinsed in 0.1 M phosphate buffer, pH 7.2 three times, 5 min each. Samples were then dehydrated in the following solutions for 5 min each: 10% ethanol, 30% ethanol, 50% ethanol, 70% ethanol, 90% ethanol, 100% ethanol (1), 100% ethanol (2), 100% ethanol (3). Sample edges were blotted during each solution change, taking care to not let samples dry out. Samples were then dried on a glass surface, coated with gold sputter coating and examined using a Zeiss Supra 55VP microscope.

### Transmission electron microscopy (TEM)

EVs isolated from conditioned media were fixed in 2.5% glutaraldehyde with 4% paraformaldehyde (EM grade) for 2.5 h and washed in PBS for 24 h. Cells were post-fixed in osmium tetraoxide for 30 min, washed with distilled water and subsequently dehydrated using increasing ethanol concentrations (70%, 85%, 95% and 100%), each for 10 min. Sample dehydration was followed by immersion in propylene for 20 min twice. Samples were infiltrated with a 1:1 mixture of propylene oxide and Spurr’s Resin for 1 h, left in 100% Spurr’s Resin overnight. Samples were then embedded in beem capsules using fresh Spurr’s Resin at 70 °C for polymerization. Excess resin was trimmed and 90 nm sections of samples were made using a Leica Ultramicrotome. Sections were placed on 200 mesh copper grids, stained with saturated uranyl acetate in 50% ethanol for 6 min, rinsed in water and stained for 90 s in lead citrate. Grids were then rinsed in water, dried on filter paper and viewed under a Fei Tecnai transmission electron microscope operated at 80 kV. Images were obtained using an AMT camera with AMT digital software.

### Preparation of EVs for immunogold electron microscopy

EVs were isolated from 25 ml of culture supernatant using differential centrifugation, permeabilized and blocked in filtered 1% bovine serum albumin (BSA) with 0.05% Triton-X in PBS, pH 7.4 for 1 h at RT. Anti-CD63 (1:100 rabbit anti-mouse antibody; Santa Cruz USA) was applied for 24 h at 4 °C. After 4 washes in PBS, a 1:50 dilution of 2 nm goat anti-rabbit antibody was applied and left for 1 h at room temperature. After 4 rinses, EVs were fixed in 4% glutaraldehyde for 2.5 h and rinsed four times over 6 h in PBS. EVs were placed in OsO4 for 1 h, rinsed 3 times over 1 h in water and then placed in an increasing ethanol dehydration series. EVs were then placed in propylene oxide for 20 min, two times, and then infiltrated for 3 h in a 1:1 propylene oxide/spurr resin mixture. EVs were left to infiltrate in pure Spurr overnight and were then embedded in Spurr overnight at 70 °C. Samples were sectioned into 90 nm slices and placed on Nickel Formvar covered 200 mesh grids. Aurion silver enhancement was brought to room temperature, 20 drops of enhancement solution was mixed with one drop of developer, and grids were left to incubate for 10 min, which enlarged the 0.5 nm diameter gold nanoparticles to 20 nm. After rinsing with water, grids were stained in 4% uranyl acetate solution, rinsed and dried. Samples were imaged using a FeiTechnai Spirit Transmission Electron Microscope operated at 80 kV and AMT digital imaging software.

### NanoSight analysis

EV size and concentration were assessed using the NanoSight NS500 system^57,107,108^. Collection of supernatant at three time points following plating, 12 h, 24 h and 48 h, was identical to isolation of EVs described above, with the exception that the supernatant was used just prior to 100, 000 × *g* ultracentrifugation for NanoSight analysis. Control media, non-conditioned, was processed under identical conditions. Based on the NanoSight protocol, to ensure accurate readings, final supernatant was diluted at 1:20 in PBS and triplicates of 1 ml samples were used for analysis. The NanoSight system uses laser to illuminate nano-scale particles, detected individually as light-scattered points moving via Brownian motion. Polydispersity was quantified, and Nanoparticle Tracking Analysis (NTA) software 2.3 used to track size and diffusion of nanoparticles. Results are displayed as a frequency size distribution graphs, describing the number of particles per ml. Significance was calculated using Student’s t-test with three independent experiments. The error bars represent standard deviation of the mean. Significant differences were denoted with asterisks: *(p < 0.05), **(p < 0.01), ***(p < 0.001), ****(p < 0.0001); “ns” indicates no significant difference.

### Sucrose gradient analysis and Western blot

EVs were analyzed using 10%- 40% sucrose (w/v) density gradient solution. A linear sucrose gradient was prepared with 12.6 ml of 10% (w/v) and 12.6 ml of 40% (w/v) sucrose solutions, mixed in a sucrose gradient device (Life technologies). An EV pellet isolated from 27 ml of conditioned medium was resuspended in 0.5 ml of PBS, loaded on top of the layered sucrose gradient and centrifuged at 18,000 × g at 4 °C for 15 h. Fractions containing EVs were harvested and the densities were determined by weighing each fixed volume. Each 1 ml fraction was then diluted in 26 ml of PBS, and ultracentrifuged for 1 h at 100,000 × g. EVs were lysed at 4 °C for 1 h in a lysis buffer containing 50 mM Tris-HCl, 1% Triton X-100, 2 mM PMSF (Sigma Aldrich), 1× Halt Protease inhibitor Cocktail (Thermo Scientific), 100 mM NaCl, 1 mM EDTA and 2 mM MgCl2 at pH7.4. Aliquots of sample lysate from each 1 ml fraction were all used for 4% to 12% Sodium Dodecyl Sulfate Polyacrylamide gel electrophoresis (SDS-PAGE) and transferred to a PVDF membrane. Protein bands were visualized with NBT/BCIP (Sigma) after membrane incubation with primary antibody anti-CD63 antibody (1:500; Rabbit Polyclonal, Santa Cruz) and secondary antibody conjugated to alkaline phosphatase (1: 10, 000; Abcam) (Figure s4).

### Mass spectrometry

Whole cell lysate and exosome enriched samples from mRPCs were denatured in 8 M urea, reduced with 10 mM DTT, and alkylated with 50 mM iodoacetamide. This was followed by proteolytic digestion with endoproteinase LysC (Wako Chemicals) overnight, and with trypsin (Promega) for 6 h at room temperature. The digestion was quenched with 2% formic acid and resulting peptide mixtures were desalted using in-house made C18 Empore (3M) StAGE tips^109^. Samples were dried and resolubilized in 2% acetonitrile and 2% formic acid. Approximately 1 μg of each sample was injected for analysis by reversed phase nano-LC-MS/MS (Ultimate 3000 coupled to a QExactive Plus, Thermo Scientific). After loading on a C18 trap column (PepMap, 5 μm particles, 100 μm × 2 cm, Thermo Scientific) peptides were separated using a 12 cm × 75 μm C18 column (3 μm particles, Nikkyo Technos Co., Ltd. Japan) at a flow rate of 200 nL/min, with a gradient increasing from 5% BufferB (0.1% formic acid in acetonitrile) / 95% Buffer A (0.1% formic acid) to 40% Buffer B / 60%Buffer A, over 140 min. All LC-MS/MS experiments were performed in data dependent mode with lock mass of m/z 445.12003^110^. Precursor mass spectra were recorded in a 300–1400m/z range at 70,000 resolution, and fragment ions at 17,500 resolution (lowest mass: m/z 100). Up to twenty precursors per cycle were selected for fragmentation and dynamic exclusion was set to 60 s. Normalized collision energy was set to 27.

### Protein profiling analysis

Mass spectrometry data were searched against a Uniprot mouse database (July 2014) using MaxQuant (version 1.5.0.30^110,111^). Oxidation of methionine and N-terminal protein acetylation were allowed as variable modifications, while all cysteines were treated as being carbamidomethylated. Precursor mass tolerance was set at 4.5 ppm while a 20 ppm tolerance was allowed for fragment ions. Two missed cleavages were allowed for specific tryptic search. The “match between runs” option was enabled. False discovery rates at the protein and peptide level were set to 1%. Protein abundances were represented by LFQ (Label Free Quantitation) and iBAQ (intensity-Based Absolute Quantitation)^112^. iBAQ values were log2(x) transformed and further used to create box plots to depict the distribution and changes in protein expression between two samples.

### RNA extraction and quantitative real-time PCR

EVs were isolated from 20 ml of media conditioned by 1 × 10^7^ mRPCs cultured in T75 flasks for 48 h and all EVs were used for RNA extraction. The same amount of EV-depleted complete control media was used as a control to verify absence of retina specific transcription factors and mouse house keeping genes in control medium. Total RNA extraction was performed using an RNeasy Mini Kit (Qiagen). Isolated RNA was measured for quality using a Nano drop ND/2000 spectrophotometer and analyzed by 2% gel electrophoresis. Prior to RNA and miRNA extraction, EVs were treated with 100 ug/ml RNAse (Thermoscientific) for 30 min at 37 °C according to manufacturer’s instruction. DNA contamination was removed using an RNase-Free DNase Set (Qiagen). cDNA was synthesized using equal amounts of RNA samples (800 ng), according to the AMV First Strand cDNA Synthesis Kit instructions (NEB Biolabs). β-actin and GAPDH were used as a housekeeping gene controls for mRNA analysis^113,114^. Quantitative real time PCR (qPCR) was performed using SYBR GreenER™ qPCR SuperMix (Life Technologies) on a Biorad system. Significance was calculated using Student’s t-test with three independent experiments. For miRNA analysis, the same amount of starting material (10 ng) from extracellular vesicles and mRPCs was used for reverse transcription. The primers used for qPCR are described in Table 1. miRNA was extracted using a MirVana RNA isolation kit (Thermo Fisher Scientific). Equal amounts of miRNA from EVs and RPCs was converted to cDNA using the TaqMan MicroRNA Reverse Transcription Kit. QPCR was performed using the Taqman Universal Master Mix II and Taqman assays (Life technologies). Analyzed miRNA assay IDs are provided in Table 2.

**Table 1:** Primers and product sizes for qRT-PCR.

Gene	Primer sequence (5′–3′)	(bp)
Nestin	F: AACTGGCACACCTCAAGATGT	235
	R: TCAAGGGTATTAGGCAAGGGG	
Sox2	F: CACAACTCGGAGATCAGCAA	190
	R: CTCCGGGAAGCGTGTACTTA	
Pax6	F: AGTGAATGGGCGGAGTTATG	132
	R: ACTTGGACGGGAACTGACAC	
Hes1	F: CCCACCTCTCTCTTCTGACG	185
	R: AGGCGCAATCCAATATGAAC	
Ki-67	F: CAGTACTCGGAATGCAGCAA	170
	R: CAGTCTTCAGGGGCTCTGTC	
GFAP	F: AGAAAACCGCATCACCATTC	184
	R: TCACATCACCACGTCCTTGT	
β-actin:	F: ACGTTGACATCCGTAAAGAC	100
	R: GCAGTAATCTCCTTCTGCAT	
GAPDH	F: ACCACAGTCCATGCCATCAC	452
	R: TCCACCACCCTGTTGCTGTA	
EGFP	F: AAGTTCATCTGCACCACCG	533
	R: TCCAGCAGGACCATGTGATCGC	
CRE	F:CTGACGGTGGGAGAATGTTAAT	271
	R:TCATCCTTAGCGCCGTAAATC

**Table 2:** miRNAs assay IDs.

*miRNAs*	*Assay IDs*
miR-182	002599
miR-96	000186
miR-204	000508
miR-9	000583
Let-7d	002283
U6 sn RNA	001973

### PKH26 labeling of EVs and uptake by mRPCs

Isolated EVs were labeled with the red fluorescent lipophilic dye PKH26 (Sigma) according to the manufacturer’s instruction. The use of fluorescence microscopy to image PKH26 labeled EVs has been demonstrated in a number of studies^67,115,116^. Briefly, EVs were collected after the 100,000 × g ultracentrifugation, and incubated in PKH26 for 5 min at room temperature. Excess dye was removed by rinsing with EV-depleted complete media followed by three rinses in PBS. EVs were re-suspended in serum-free medium and incubated with mRPCs for 24 h at 37 °C. mRPCs were cultured on coverslips coated with mouse laminin (Life Technologies, USA) 24 h before culturing with PKH26 labeled EVs. mRPCs were fixed in 4% PFA for 10 min and washed with PBS 3 times, 10 min each. Low and high power images of EV binding and internalization were captured with a Nikon confocal spinning disk microscope using a 20× or 60× objective^20,117,118^. A HeNe laser was used for PKH26-labelled EVs (551/567). A 488 Argon Laser was used to image mRPC GFP. As a control, co-localization of CD63 with PKH26 labelled EVs was analyzed by adding PKH26 labelled EVs to mRPCs for 24 h, as above. mRPCs were then rinsed three times with PBS, fixed in 4% PFA for 30 min, blocked and permeabilized for 30 min in 0.1% Triton X-100 and 3% bovine serum albumin (BSA) in PBS. Anti-CD63 (1:50; Rabbit Polyclonal, Santa Cruz) was added to mRPCs for overnight incubation at 4 °C. After rinsing three times with PBS, mRPCs were incubated with a fluorescein conjugated secondary antibody, Cyanine 5 (1:500; goat anti-rabbit IgG, ThermoFisher) for 1 h at room temperature. After rinsing once for 15 min and thrice for 5 min with wash buffer, mRPCs were mounted using ProLong Gold media with DAPI (ThermoFisher). Images were taken using a Nikon Ti microscope with a 40× objective and the fluorophores Cy5 (649/666), PKH26 (551/567) and DAPI (358/461).

### RT-PCR analysis of GFP mRNA EV content transfer between mRPCs and hRPCs

The transwell system was used for EV content transfer between GFP+ mRPCs and non-GFP hRPCs. The use of a transwell culture system to evaluate EV mediated transfer of miRNA and mRNA between cell populations has been successful in a number of published studies^119–121^. Briefly, 5 × 10^5^ GFP+ mRPCs were plated on transwell inserts (0.4 µm pore size) above non-GFP hRPCs in each 6-well plate; the same number of hRPCs in plate, without mRPCs, were used as negative control. Cells were grown at 37 °C and 5% CO_2_ in neural basal complete media as above, containing 2 mM glutamate, 1× EV-depleted B27, 1× N2 supplement, 20 ng/ml recombinant epidermal growth factor (rEGF), 10 ng/ml rhbFGF, 50 µg/ml nystatin, penicillin-streptomycin (10 IU ml^−1^ and 20 µg ml^−1^, respectively. hRPCs were collected after 96 h, rinsed using PBS and processed for mRNA extraction.

### Fluorescence analysis of Cre EV content transfer between mRPCs

P0 mouse retina were isolated and electroporated with pCAG-Cre plasmid and pCALNL-GFP plasmid as described previously^122^. The pCAG-Cre and pCALNL-GFP plasmids were gifts from Connie Cepko (Addgene plasmids #13775 and 13770)^68^. For electroporation efficiency, P0 retina were electroporated and then dissociated into mRPCs for analysis. Briefly, retina were placed into an electroporation chamber filled with plasmids/1× PBS and 5 pules of 25 volts were applied to drive plasmids into retinas. Electroporated retina were placed on a polycarbonate filter (0.2 μm pore size) for 24 h in media containing 45% DMEM, 45% Ham’s F12 Nutrient Mixture (Life Technologies), 10% fetal calf serum, L-glutamine (2 mM) penicillin (100 U/ml), and streptomycin (0.1 mg/ml) and then manually dissociated into mRPCs. Electroporation efficiency was determined by counting the number of GFP positive mRPCs dissociated from retina that were electroporated with both plasmids. Dissociated mRPCs with or without pCAG-Cre were cultured in transwells with 0.4 µm pores and dissociated retina cells containing the LoxP plasmid were seeded in 6 well plates for 3 weeks. Images were taken in phase and FITC every other day at 20× using an EVOS FLoid Cell Imaging Station (Thermofisher). GFP expressing reporter^+^ cells were counted from n = 3 experimental and n = 3 control wells and intensity quantified using ImageJ. Significance was calculated using Student’s t-test with three independent experiments. The error bars represent standard deviation of the mean. Significant differences were denoted with asterisks: *(p < 0.05), **(p < 0.01), ***(p < 0.001), ****(p < 0.0001); “ns” indicates no significant difference.

This work was made possible due to grants from the National Institute On Minority Health and Health Disparities M.E. (3G12MD007603-30S2), the National Institute of General Medical Sciences S.R. (5SC3GM113782) and the National Eye Institute S.R. (5R21EY026752-02).

## Electronic supplementary material

Nanosight imaging of mRPC released EVs

S1-S6

S7
