# Author Correction: Retinal progenitor cells release extracellular vesicles containing developmental transcription factors, microRNA and membrane proteins

**DOI:** 10.1038/s41598-018-32118-6

**Published:** 2018-10-22

**Authors:** Jing Zhou, Alberto Benito-Martin, Jason Mighty, Lynne Chang, Shima Ghoroghi, Hao Wu, Madeline Wong, Sara Guariglia, Petr Baranov, Michael Young, Rajendra Gharbaran, Mark Emerson, Milica Tesic Mark, Henrik Molina, M. Valeria Canto-Soler, Hector Peinado Selgas, Stephen Redenti

**Affiliations:** 1 Department of Biological Sciences, Lehman College, City University of New York, 250 Bedford Park Boulevard West, Bronx, NY 10468 USA; 2 Biology Doctoral Program, The Graduate School and University Center, City University of New York, 365 5th Avenue, New York, NY 10016 USA; 3 Children’s Cancer and Blood Foundation Laboratories, Departments of Pediatrics, and Cell and Developmental Biology, Drukier Institute for Children’s Health, Meyer Cancer Center, Weill Cornell Medical College, New York, New York 10021 USA; 4 Nikon Instruments Inc, 1300 Walt Whitman Road, Melville, NY 11747 USA; 5 Department of Environmental Health Sciences, Mailman School of Public Health, Columbia University, 722 West 168th St, New York, NY 10032 USA; 6 The Schepens Eye Research Institute, Massachusetts Eye and Ear, Harvard Medical School, 20 Staniford Street, Boston, MA 02114 USA; 7 Department of Biology, The City College of New York, City University of New York, New York, NY 10031 USA; 8 Proteomics Resource Center, The Rockefeller University, 1230 York Avenue, New York, NY 10065 USA; 9 The Wilmer Eye Institute, Johns Hopkins University School of Medicine, Baltimore, MD 21287 USA; 10 Microenvironment and Metastasis Laboratory, Department of Molecular Oncology, Spanish National Cancer Research Centre (CNIO), Melchor Fernández Almagro, 3, Madrid, E28029 Spain; 11 Biochemistry Doctoral Program, The Graduate School and University Center, City University of New York, 365 5th Avenue, New York, NY 10016 USA

Correction to: *Scientific Reports* 10.1038/s41598-018-20421-1, published online 12 February 2018

The original version of this Article contained a typographical error in the spelling of the author M. Valeria Canto-Soler, which was incorrectly given as M. Valeria Canto-Solar. This has now been corrected in the PDF and HTML versions of the Article and in the accompanying supplementary material.

