# Supplementary material for: Retinal progenitor cells release extracellular vesicles containing developmental transcription factors, microRNA and membrane proteins
Source: Sci Rep. 2018 Feb 12;8:2823. doi: 10.1038/s41598-018-20421-1 (PMC5809580; doi:10.1038/s41598-018-20421-1)
Supplement: Supplementary file 2 — S1-S6 [file 41598_2018_20421_MOESM2_ESM.doc]

**Retinal progenitor cells release extracellular vesicles containing developmental transcription factors, microRNA and membrane proteins**

Jing Zhou^1,2^, Alberto Benito-Martin^3^, Jason Mighty^1,2^, Lynne Chang^4^, Shima Ghoroghi^1^, Hao Wu^1,2^, Madeline Wong^1^, Sara Guariglia^5^, Petr Baranov^6^, Michael Young^6^, Rajendra Gharbaran^1^, Mark Emerson^2,7^, Milica Tesic Mark^8^, Henrik Molina^8^, M. Valeria Canto-Soler^9^ Hector Peinado Selgas^3,10^ and Stephen Redenti^1,2,11^

**Supplementary Video:**

**Title: Nanosight imaging of mRPC released EVs**

**Legend:** A sample of Nanosight video data showing laser diffraction of mRPC EVs released into media over 24 h. EVs exhibit Brownian motion and observable trajectories, which can be analyzed to determine hydrodynamic diameters and concentrations.

**Supplementary Figures:**


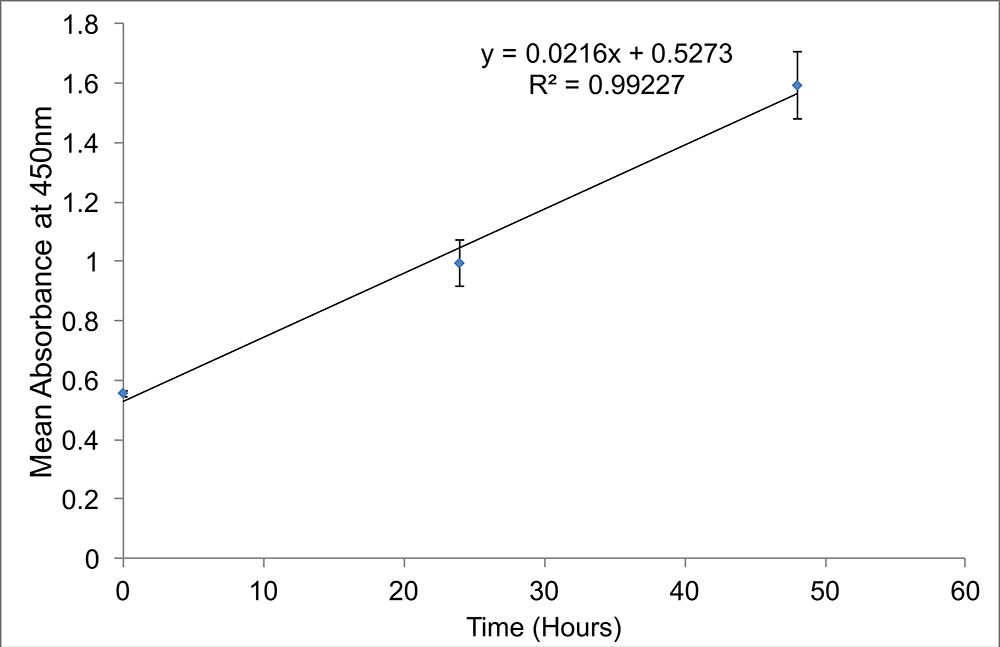


**Title: Supplementary Figure S1: WST viability and proliferation activity of mRPCs up to 48 h in culture.**

**Legend:** To verify viability mRPCs were plated at a concentration of 1.0*10^5^cells/cm^3^ in 24 well plates and analyzed at 0, 24 and 48 h. WST-1 is a colorimetric assay that uses a soluble tetrazolium salt, which is then cleaved to a colored product known as formazan by the reductase system of metabolically live cells. The graph shows a positive linear slope indicating that the mRPCs were viable and proliferating during experimental time points.


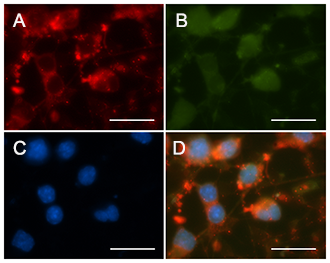


**Title: Supplementary Figure S2. Low magnification microscopic image of PKH26 labeled EVs adherent to mRPCs.**

**Legend:** Under low power (20X) microscopy, PKH26 labeled EVs are imaged widely distributed and binding to mRPCs in culture. A) EVs isolated from mRPCs labeled with the red PKH26 fuse to mRPCs. B) GFP^+^ mRPCs remain viable in culture expressing GFP, C) Nuclei, D) Overlay of A–C. scale 100 μm.


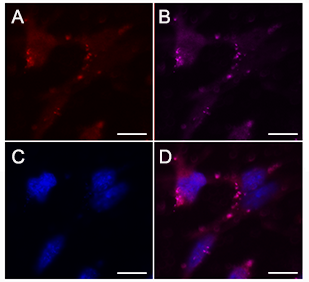


**Title: Supplementary Figure S3. mRPC EV PKH26 and CD63 co-localization in target mRPCs.**

**Legend:** EVs labeled with PKH26 were internalized by target mRPCs and exhibit co-localization of the exosome marker CD63. A) PKH26 labeled mRPC EVs are internalized by mRPCs, B) CD63 labeling localizes to EVs, C) nuclei and D) Overlay of A-C. Scale 25 μm


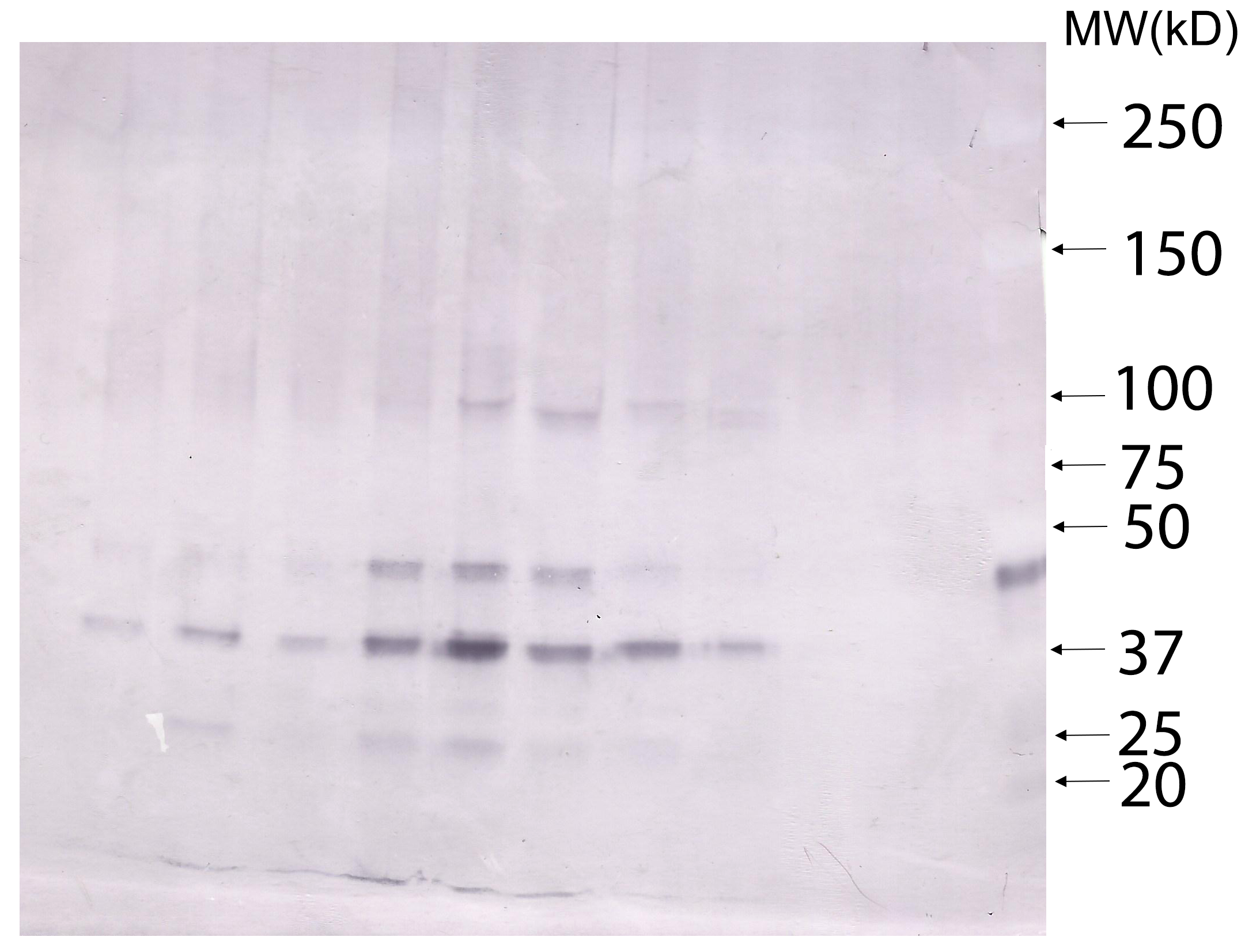


**Title: Supplementary Figure S4. Full Gel Blot from Figure 3. Sucrose-gradient and western blot characterization of extracellular vesicles.**

**Legend:** The sucrose gradient band revealing CD63 positive bands detected in fractions at gradient-densities of 1.12-1.17g/cm^3^ was cropped from the above blot.

**Supplementary Tables:**

**Title: Proteomic Dataset Tables of mRPC EV Cargo**

**Legend:** Proteomics results were organized into tables describing EV genesis and structure: Table S1. RabGTPase proteins and Table S2. Proteins of the ESCRT complex. As well as proteins involved in neural development and function: Table S3. Neural multipotentcy and developmental proteins, Table S4. Transport and channel proteins, and Table S5. Na +/K+-ATPase subunits.

|  | **Table S1. RabGTPase proteins** |  |  |
| --- | --- | --- | --- |
| **Gene name** | **Protein name** | **LOG2 DIFF** | **Razor + EV Peptides** |
| Rala | Ras-related protein Ral-A | Exo | 2 |
| Hras1 | GTPaseNRas | Exo | 1 |
| Rab2a | Ras-related protein Rap-2a | Exo | 1 |
| Rab3d | Ras-related protein Rab-3D | Exo | 1 |
| Rras2 | Ras-related protein R-Ras2 | Exo | 2 |
| Rab13 | Ras-related protein Rab-13 | Exo | 1 |
| Rab4b | Ras-related protein Rab-4B | Exo | 1 |
| Rab22a | Ras-related protein Rab-22A | Exo | 2 |
| Rab21 | Ras-related protein Rab-21 | Exo | 1 |
| Rap2c | Ras-related protein Rap-2c | 5.12835 | 13 |
| Rap2b | Ras-related protein Rap-2b | 4.62015 | 4 |
| Rab35 | Ras-related protein Rab-35 | 4.36185 | 8 |
| Rab31 | Ras-related protein Rab-31 | 4.02845 | 5 |
| Rab8A | Ras-related protein Rab-8A | 3.71465 | 6 |
| Rab23 | Ras-related protein Rab-23 | 3.69175 | 3 |
| Rras | Ras-related protein R-Ras | 3.63525 | 6 |
| Rap1b | Ras-related protein Rap-1b | 3.62735 | 10 |
| Rab8b | Ras-related protein Rab-8B | 3.42905 | 1 |
| Rab10 | Ras-related protein Rab-10 | 2.98525 | 7 |
| Ralb | Ras-related protein Ral-B | 2.87535 | 10 |
| Rasa3 | RasGTPase-activating protein 3 | 2.63755 | 14 |
| Rac1; Rac3 | Ras-related C3 botulinum toxin substrate 1 | 2.30775 | 8 |
| Rab1;Rab1A | Ras-related protein Rab-1A | 2.25885 | 10 |
| Rab6a;Rab6b | Ras-related protein Rab-6A; Rab-6B | 1.60205 | 6 |
| Rab5c | Ras-related protein Rab-5C | 1.56565 | 7 |
| Rab7a | Ras-related protein Rab-7a | 1.47565 | 13 |
| Rab12 | Ras-related protein Rab-12 | 1.33805 | 1 |
| Rap1a | Ras-related protein Rap-1A | 1.26375 | 1 |
| Rab2a;Rab2b | Ras-related protein Rab-2A; Rab-1B | 1.24335 | 7 |
| Rab11a;Rab11b | Ras-related protein Rab-11A | 1.07785 | 10 |
| Rab14 | Ras-related protein Rab-14 | 0.83715 | 5 |
| Rab5a | Ras-related protein Rab-5A | 0.75265 | 1 |
| Rab-9A;Rab-9 | Ras-related protein Rab-9A | 0.72285 | 1 |
| Anxa4 | Annexin A4 | Exo | 7 |
| Anxa6 | Annexin;Annexin A6 | Exo | 7 |
| Anxa7 | Annexin A7 | 3.53235 | 4 |
| Anxa2 | Annexin A2 | 0.89295 | 24 |
| Anxa5 | Annexin A5 | 0.82925 | 20 |

|  | **Table S2. Proteins of the ESCRT complex** |  |  |  |
| --- | --- | --- | --- | --- |
| **Gene name** | **Protein name** | **LOG2 DIFF** | **Razor + EV Peptides** | **ESCRT complex** |
| Hgs | Hepatocyte growth factor-regulated tyrosine kinase substrate | 0.06805 | 6 | ESCRT-0 |
| Vps37c | Vacuolar protein sorting-associated protein 37C | Exo | 5 | ESCRT-I |
| TSG101(Vps23) | Tumor susceptibility gene 101 protein | 4.93825 | 20 | ESCRT-I |
| Vps37b | Vacuolar protein sorting-associated protein 37b | 8.01345 | 15 | ESCRT-I |
| Vps28 | Vacuolar protein sorting-associated protein 10 | 4.03305 | 10 | ESCRT-I |
| Vps36 | Vacuolar protein sorting-associated protein 36 | 3.53475 | 17 | ESCRT-II |
| Vps25 | Vacuolar protein sorting-associated protein25 | 3.00285 | 8 | ESCRT-II |
| Snf8(Vps22) | Vacuolar-sorting protein SNF8 | Exo | 5 | ESCRT-II |
| Vps4a | Vacuolar protein sorting-associated protein 10 | 3.95815 | 4 | ATPase complex |
| Vps4b | Vacuolar protein sorting-associated protein4b | 1.95295 | 14 | ATPase complex |
| Pdcd6ip | Programmed cell death 6-interacting protein | 7.37565 | 51 | Accessory |
| Chmp6 | Charged multivesicular body protein 6 | Exo | 3 | ESCRT-III |
| Chmp2b | Charged multivesicular body protein 2b | Exo | 1 | ESCRT-III |
| Chmp3 | Charged multivesicular  body protein 3 | 2.54505 | 3 | ESCRT-III |
| Chmp1a | Charged multivesicular  body protein 1a | 1.90215 | 7 | ESCRT-III |
| Chmp1b2 | Charged multivesicular  body protein 1b-1 and2 | 1.59615 | 1 | ESCRT-III |

|  | **Table S3. Neural multipotentcy and developmental proteins** |  |  |  |
| --- | --- | --- | --- | --- |
| **Gene name** | **Protein name** | **LOG2 DIFF** | **Razor + EV Peptides** | **Function** |
| Adam10 | Disintegrin and metalloproteinase domain-containing protein 10 | 5.94535 | 10 | 23129104 : maintain progenitor cell pool, Controls Notc |
| Klf14 | Krueppel-like factor 14 | Exo | 1 | Master regulator of gene expression |
| Pxdn | Peroxidasin homolog | 4.92205 | 10 | 24895407 cell proliferation, differentiation and eye basement membrane consolidation |
| Itga6 | Integrin alpha6;Integrin alpha-6 heavychain;Integrin alpha-6 light chain | 4.72065 | 24 | 9742403retinal lamination, neurite outgrowth |
| Slc16a1 | Monocarboxylate transporter 1 | 4.68095 | 2 | Müller and plexiform layers, lactate exchange between neurons and glia,neural metabolism |
| Ntn1 | Netrin-1 | 4.56635 | 15 | 10843775 :axonpathfinding |
| Gpm6a | Neuronal membrane glycoprotein M6-a | 4.53985 | 4 | neuronal differentiation, migration, neurite outgrowth |
| Lphn3 | Latrophilin-3 | 4.39535 | 4 | 24273166 :cell adhesion, synapse formation |
| Gpm6b | Neuronal membrane glycoprotein M6-b | 4.34355 | 8 | membrane strutural protein |
| Prelp | Prolargin | 4.069 | 10 | 23074202 :retina differentiation, proliferation, migration, axon guidance |
| Atp2b1 | ATPase, Ca++ transporting, plasma membrane | 4.03085 | 16 | neural retina initial formation to mature structure |
| Ano6 | Anoctamin-6 |  |  | photoreceptor Calcium (Ca2+)-activated chloride (Cl−) channels |
| Slc12a2 | Solute carrier family 12 member 2 |  |  | Distal synapses and synaptogenesis in mouse retinas |
| Bsg | Basigin | 3.74485 | 5 | 11853760 :effects retinal development |
| Kras | GTPaseKRas;GTPaseKRas, N-terminally processed | 3.54895 | 5 | 21576358 :Kras signaling not only rescued the retinal cell numbers |
| Pabpc4;Gm10110 |  | 2.96415 | 12 | 24469397 :mRNA stability, protect from decay, expression of mRNA |
| Med23 | Mediator of RNA polymerase II transcription subunit 23 | Exo | 1 | Required for transcriptional activation, assembly of the pre-initiation complex |
| Ncam1 | Neural cell adhesion molecule 1 | 2.82135 | 1 | 7402293 :histogenesis of the developing retina |
| Arf6 | ADP-ribosylation factor 6 | 2.81815 | 5 | 12686588 :control of membrane trafficking during neuritogenesis |
| Serpine2 | Glia-derived nexin | 2.78505 | 21 | 2335138 :protease inhibitor, dendrite length |
| Pabpc1 | Polyadenylate-binding protein 1 | 2.52565 | 30 | regulatory processes of mRNA metabolism such as pre-mRNA splicing |
| Gpr56 | G-protein coupled receptor 56;GPR56 N-terminal fragment;GPR56 C-terminal fragment | 2.50025 | 3 | 21768377 :development, retinal lamination |
| Epb41l5 | Band 4.1-like protein 5 | 2.35755 | 3 | 18373558 :GluR8 mediated signal |
| Egfr | Epidermal growth factor receptor | 1.88155 | 10 | progenitor proliferation |

|  | **Table S4. Transport and channel proteins** |  |  |
| --- | --- | --- | --- |
| **Gene name** | **Protein name** | **LOG2 DIFF** | **Razor + EV Peptides** |
| Slc7a1 | High affinity cationic amino acid transporter 1 | Exo | 4 |
| Slc1a4 | Neutral amino acid transporter A | Exo | 2 |
| Slc30a | Zinc transporter 1 | Exo | 3 |
| Slc2a1 | Solute carrier family 2, facilitated glucose transporter member 1 | Exo | 1 |
| Slc44a | Choline transporter-like protein 1 | Exo | 2 |
| Slc39a | Zinc transporter ZIP10 | Exo | 4 |
| Slc1a2 | Excitatory amino acid transporter 2 | Exo | 2 |
| Slc6a1 | Sodium- and chloride-dependent GABA transporter 1 | Exo | 1 |
| Slc4a7 | Sodium bicarbonate cotransporter 3 | Exo | 1 |
| Cnnm1 | Metal transporter CNNM1 | Exo | 1 |
| Slc7a5 | Large neutral amino acids transporter small subunit 1 | 5.41155 | 6 |
| Slc16a | Monocarboxylate transporter 1 | 4.68095 | 6 |
| Slc39a | Zinc transporter ZIP6 | 3.05505 | 2 |
| Cacng7 | Voltage-dependent calcium channel gamma-7 subunit | Exo | 1 |
| Cacna2d1 | Voltage-dependent calcium channel subunit alpha2/delta-1; | Exo | 3 |
| Ttyh2 | Protein tweety homolog 2 | Exo | 3 |
| Ttyh3 | Protein tweety homolog 3 | Exo | 7 |
| Clic1 | Chloride intracellular channel protein 1 | 4.03085 | 8 |
| Ano6 | Anoctamin-6 | 3.92735 | 6 |

|  | **Table S5. Na +/K+-ATPase subunits** |  |  |
| --- | --- | --- | --- |
| **Gene name** | **Protein name** | **LOG2 DIFF** | **Razor + EV Peptides** |
| Atp1a1 | Sodium/potassium-transporting ATPase subunit alpha-1 | 4.02965 | 25 |
| Atp1a2;Atp1a3 | Sodium/potassium-transporting ATPase subunit alpha-2 and 3 | Exo | 2 |
| Atp1a4 | Sodium/potassium-transporting ATPase subunit alpha-4 | Exo | 1 |
| Atp1b2 | Sodium/potassium-transporting ATPase subunit beta-2 | Exo | 2 |
| Atp1b1 | Sodium/potassium-transporting ATPase subunit beta-1 | Exo | 2 |
| Atp1b3 | Sodium/potassium-transporting ATPase subunit beta-3 | 2.34675 | 4 |

|  | GAPDH | β-actin | GFP | GFAP | Hes1 | Ki67 | Nestin | Pax6 | Sox2 |
| --- | --- | --- | --- | --- | --- | --- | --- | --- | --- |
| Exo-depleted control media | NA | NA | NA | NA | NA | NA | 37.37 | NA | 37.41 |
| Exo-depleted control media | NA | NA | NA | NA | NA | NA | 37.22 | NA | 38.18 |
| Exo-depleted control media | NA | NA | NA | 38.90 | NA | NA | NA | NA | NA |
| mRPC | 19.19 |  |  |  |  |  |  |  |  |
| mRPC | 19.22 |  |  |  |  |  |  |  |  |
| mRPC | 19.17 |  |  |  |  |  |  |  |  |

**Title: Supplementary Table S6. Control qPCR of exo-depleted control media.**

**Legend:** qPCR of exo-depleted control media reveals that the RNA reported in mRPC EVs were not present in control media. qPCR results show Ct numbers for exo-depleted control medium at either > 37 or NA, indicating the absence of mouse gene signal. EVs isolated from 20ml of exo-depleted control medium was used for RNA extraction. mRPC cDNA was used as a positive control.

**Title: Supplementary Table S7. Full Proteomic Data Set**.

**Legend:** In this table, proteins are marked as potential extracellular vesicle proteins (marked by +) if they were only measured in the Extracellular vesicle enriched sample or if the normalized intensity based absolute quantitation measure (iBAQ) was 8 fold higher in the Extracellular vesicle enriched sample. In separate columns are marked published exosome marker proteins (+) in addition to proteins that can act as markers for nuclear and mitochondrial contaminations.
